# Diagnostic predictive values of the Hain genotype MTBDRsl assay in mycobacterial strains isolated from Sudan

**DOI:** 10.11604/pamj.2019.32.124.12762

**Published:** 2019-03-15

**Authors:** Muatsim Ahmed Mohammed Adam, Hamdan Mustafa Hamdan Ali, Eltahir Awad Gasim Khalil

**Affiliations:** 1 National Public Health Laboratory, National Tuberculosis Reference Laboratory, Federal Ministry of Health, Khartoum, Sudan; 2 National Tuberculosis Control Program, Federal Ministry of Health, Khartoum, Sudan; 3 Department of Clinical Pathology & Immunology, Institute of Endemic Diseases, University of Khartoum, Khartoum, Sudan

**Keywords:** Hain genotype MTBDRsl, MDR, XDR, Sudan, tuberculosis

## Abstract

**Introduction:**

hain GenoType MTBDRsl is nucleic acid amplification assay based on reverse hybridization with specific oligonucleotide probes on nitrocellulose strips. MTBDRsl identifies *M. tuberculosis* complex and detects resistance to fluoroquinolone, second line injectable drugs and ethambutol evident as mutations of *gyrA, rrs and embB* genes respectively. This study aimed to evaluate the diagnostic performance of the Hain GenoType MTBDRsl Assay using 1% proportion method on LJ medium as gold standard.

**Methods:**

a total of 52 rifampicin resistant (RR) isolates were tested for second line drug sensitivity by 1% proportion method and by MTBDRsl assay.

**Results:**

two strains were identified as mycobacteria other than tuberculosis MOTT and the rest were *Mycobacterium tuberculosis* complex MTBC. Five of the MTBC isolates (5/50; 10%) showed resistance to at least one second line drug and one isolate (1/50; 2%) was XDR. XDR strain was concordantly detected by the two methods. One of two Kanamycin-resistant isolates showed discordant results. Ofloxacin showed one false positive and one false negative result. Most discrepancies were detected with Ethambutol. The sensitivity, specificity, positive and negative predictive values were respectively as follows: Ethambutol (63.3.4%, 85.7%, 94.4% and 62%), for Kanamycin (67%, 100%, 100% and 97.9%), for Amikacin and Capreomycin (100%, 100%, 100% and 100%), for Ofloxacin (75%, 97.5%, 75% and 97.8%). For XDR isolate the values were 100%, 100%, 100% and 100% respectively.

**Conclusion:**

MTBDRsl showed high specificity and negative predictive values making it acceptable and time-saving for early presumptive detection of resistance to second-line drugs in Sudan.

## Introduction

Drug resistant TB has emerged as a significant global public health problem and a great challenge for TB control. Drug resistant TB has resulted from drug misuse and/or drug mismanagement. In 2014 WHO estimate 480 000 multi drug resistant (MDR) cases; Most of these cases are in India, China, Russian Federation and South Africa [1]. Extensively drug-resistant TB (XDR-TB) had been reported by 105 countries by 2015. The magnitude of XDR-TB is not small (9.7% of MDR-TB) in addition XDR-TB is an important killer of TB patients [1, 2]. Most species of mycobacteria grow slowly with generation time of 18 to 24 hours; resulting in delay of TB diagnosis by conventional methods [3]. Molecular techniques reducing turnaround time (TAT) required to detect and identify *M. tuberculosis* to 1-2 days instead of several weeks in the conventional methods [4]. Extensively drug-resistant tuberculosis (XDR-TB) is defined as multi drug resistant tuberculosis MDR-TB; resistance of rifampicin and isoniazid with additional resistance to any fluoroquinolone and to at least one of three injectable drugs used for TB treatment: capreomycin, kanamycin, or amikacin [5]. In recent years several molecular methods have been developed for the diagnosis of tuberculosis and detection of drug resistance, including line probe assays [6]. Line probe assays (LPAs) are not new; nevertheless Hain Lifescience (Hain Lifescience GmbH, Germany) developed two kits of line probe assay for the detection of drug resistance, the genotype MTBDRplus and MTBDRsl [7] the later is one of the few commercially available molecular tests for detection of second line drugs [8]. MTBDRplus simultaneously detects MDR and the MTBDRsl detect the resistance of aminoglycosides/cyclic peptides, fluoroquinolones, and ethambutol through detection of mutations in the relevant genes [7]. In 2015, the Hain Lifescience developed the commercially available version 2.0 of the MTBDRsl assay and recently recommended by WHO [1]; to date version 2.0 MTBDRsl is not supplied in Sudan. To our knowledge; in Sudan second line drug resistance and XDR susceptibility testing has never been studied before this report.

## Methods

**Isolation of mycobacteria:** Lowenstein-Jensen medium (L.J.) containing glycerol and Lowenstein-Jensen supplemented with pyruvate were used for isolation of mycobacteria from sputum specimens. Petroff method for decontamination (4% NaOH for 20 minutes) was used to homogenize and decontaminate sputum specimens.

**Isolates identification:** isolates were firstly identified phenotypically according to their reaction with ZN staining method, growth rate, colonial morphology and pigment production and by their susceptibility to Para nitro benzoic acid (PNB). Then isolates were identified genetically by the hybridization of isolates DNA Amplicons with TUB probe incorporated on validity zone within the Hain GenoType MTBDRsl strips.

**1% proportion method:** DST was performed following the standard 1% proportion method. Second line anti-TB drugs were added into LJ media in different critical concentrations as follows: Kanamycin (KM), 30μg/ml; Capreomycin (CM), 40μg/ml; Ofloxacin (Ofx), 2.0μg/ml [9] and Amikacin (AM) 40μg/ml [10]. First line Ethambutol (EMB) was added to reach the final critical concentration of 2.0μg/ml [9].

**Figure 1:**
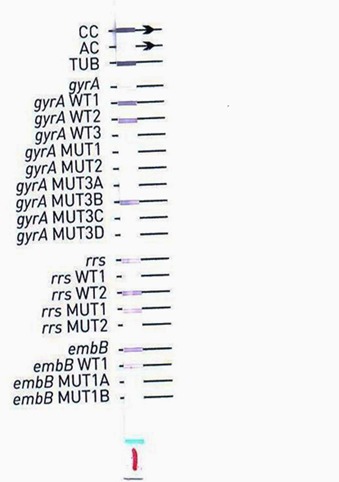
manual reading of hybridized probes of XDR using the manufacture reading sheet; CC: conjugate control; AC: amplification control; TUB: *M. tuberculosis complex*; WT: wild type probe and MUT: mutation probe

**Hain GenoType MTBDRsl Assay:** DNA extraction, amplification, hybridization and band detection were performed according to Hain GenoType MTBDRsl commercial kits (Hain life science 2009) [11]. Interpretation of the strips can be performed manually with evaluation sheet provided with the manufacture kit set. The evaluation sheet contains 22 reaction zones, three of them for the test validity (CC, AC, TUB); 10 for fluoroquinolones *(gyrA)*; 5 for injectable drugs *(rrs)* and the rest for ethambutol *(emb)* (Figure 1). When all wild type (WT) probes of the gene stain positive there is no detectable mutation within the examined region this indicates that the strain is sensitive for the respective drug. The mutation probes (MUT) some of the most common resistance-mediating mutations, the banding pattern obtain concluded about a resistance to the tested strain. Alternatively interpretation of strips performed by Genoscan system; which was designed specifically for quick and reliable results interpretation of the genotype test strips. The strips are scanned while still in the incubation tray. An image of the scanned strip is displayed directly on the screen; the software then performs a fast and objective analysis of the strips. The results are electronically saved and transmitted to the laboratory information technology (IT) system. Presence of band on (*gyrA* MUT3B) shows fluoroquinolone resistance; Absences of bands on (*rrs* WT1; and presence of *rrs* MUT2) shows resistance of injectable drugs. CC: conjugate control; AC: amplification control; TUB: *M. tuberculosis complex*; WT: wild type probe and MUT: mutation probe.

**Data handling and data analysis:** Epi Info statistical program version 3.5.1 august 13, 2008 was used to calculate the statistical significance by calculation of p. value, Odds ratio and Relative risk. Sensitivity, specificity, positive predictive value and negative predictive value of the Hain GenoType MTBDRsl were calculated according to the following equations: when the conventional 1% proportion method as the gold standard method whereas T: true; F: false; P: positive; N: negative.

**Sensitivity**:

$$=\frac{TP}{TP+TN}\%$$

**Specificity:**

$$=\frac{TN}{FP+TN}\%$$

**Positive predictive value (PPV):**

$$=\frac{TP}{TP+FP}\%$$

**Negative predictive value (NPV):**

$$=\frac{TP}{TP+FP}\%$$

## Results

Hain Genotype MTBDRsl showed significantly reduced sensitivity for Kanamycin (p = 0.04) and Ofloxacin (p = 0.00; Odds ratio: 135; Relative risk: 34.5) at 67% and 75% respectively (Table 1). For the first line drug Ethambutol the specificity was 85.7%. Sensitivity was high (p = 0.00; Odds ratio 15.55; Relative risk 6.33). Sensitivity and specificity were higher for Amikacin and Capreomycin at 100% and 100% respectively. The sensitivity was also higher with statistical correlation (p = 0.02) for XDR and for strain identification at 100%. Two strains out of the total (2/52) 3.8 % were identified as rifampicin resistant (RR) Mycobacterium other than tuberculosis MOTT; and the rest were multi drug resistant (MDR) *Mycobacterium tuberculosis* complex MTBC (50/52) 96.2% (Table 2). MOTT isolates were concordantly detected by the 1% proportion method and Hain GenoType MTBDRsl.

**Table 1:** diagnostic performance of MTBDR*sl* compared to 1% proportion method as a reference standard

Test	Sensitivity (%)	Specificity (%)	Positive predictive value (%)	Negative predictive value (%)
XDR	100	100	100	100
AM	100	100	100	100
KM	67	100	100	97.9
CM	100	100	100	100
Ofx	75	97.8	75	97.8
EMB	90.4	60	63.3	90
Strain identification	100	100	100	100

**Table 2:** comparison of Hain MTBDR*sl* assay and 1% proportion method as a reference standard

Test	MTBDR*sl* assay			1% proportion method		
	R	S	Total	R	S	Total
XDR	1	49	50	1	49	50
AM	1	49	50	1	49	50
KM	1	49	50	2	48	50
CM	1	49	50	1	49	50
Ofx	4	46	50	4	46	50
EMB	20	30	50	30	20	50
MOTT	-	-	2	-	-	2

## Discussion

**Strain identification:** hain GenoType MTBDRsl performance in strain identification was excellent; sensitivity, specificity and predictive values (PVs) for strain identification were much higher 100% for each; MOTT was reported at a low rate in our samples, as expected this bacteria originates from the soil and water and infects patients with chronic pulmonary disease or immune-deficient patients [12]. Accuracy of strain identification is an additional advantage for the Hain GenoType MTBDRsl assay; similar results were reported in Africa and South America [13].

**Injectable second line drugs:** different from kanamycin; high rates of sensitivity and specificity (100% each; p=0.02) for Amikacin were also reported before by Kontsevaya *et al* in 2012 [14]. Similarly, for Capreomycin sensitivity of 100% and specificity of 86.4% were acceptably high for our cohort was better than that reported elsewhere [14-17]. The small sample size has to be taken into consideration. Performance of Hain GenoType MTBDRsl was reduced for Kanamycin; sensitivity (67%) and the very high specificity (100%) (p=0.04) reported for Kanamycin may be attributed to a lesser extent to its wide use in the treatment of TB, compared to Amikacin and Capreomycin due to the cost issues. In spite of the small number, Kanamycin resistant isolates discrepancy was noted in the result, this can be attributed to new mutations not incorporated in the test strip. Moreover, this can reflect the study limitation that can be solved by sequencing. Similar results have been reported from different parts of the world [14]. This indicates that second line injectable drugs have no superiority over each other. The lower cost of Kanamycin makes it preferable by some [18]. The reported low frequencies of resistance to injectable anti-TB drugs are similar to that reported by different parts of the world [19].

**Fluoroquinolones (Ofloxacin):** low sensitivity of Hain GenoType MTBDRsl in picking up resistance to Ofloxacin (75%) was not matched to the remarkably high specificity (97.8%) that was reported (p=0.00); Positive predictive value and negative predictive value (75%) and (97.8%) respectively. Although the number of Ofloxacin resistant isolates is small, discordance was seen where one false positive and false negative result were reported. This can be attributed to missed mutation that is not involved in the test strip in addition to probable cross resistance. Low sensitivity and high specificity for fluoroquinolones has been previously reported [15, 20].

**XDR:** the majority of MDR (90%, 45/50) were susceptible to the all second line anti-tuberculous drugs. MTBDRsl showed very high performance (p=0.02) in detecting XDR with sensitivity of (100%) and specificity of (100%). Positive and negative predictive values were (100%) and (100%). The isolate was concordantly detected by the two methods Table1. To our knowledge; in Sudan second line drug resistance and XDR susceptibility testing has never been studied before this report. The low frequency of XDR-TB within the study cohort is within the reported frequency that was reported from many countries (0.8-15.2%) [19]. but is also this study constrained by the limited sample size.

**First line Ethambutol:** the specificity (85.7%) of first line Ethambutol (p = 0.00; Odds ratio 15.55; Relative risk 6.33) positive predictive value and negative predictive values (90.4%) and (62%) respectively; points to the fact that even in conventional methods the interpretation of drug sensitivity testing (DST) to some of the first- and second-line drugs is difficult such as ethambutol. This indicates that, the accuracy of DST of ethambutol is less reliable and reproducible and it is questionable as was shown before [18]. Moreover; this may reflect the imperfect reference standard currently available for this drug [17, 20].

## Conclusion

In conclusion Hain GenoType MTBDRsl showed high specificity and negative predictive values (NPVs) making it an acceptable, simple and time-saving technique for early presumptive detection of resistance to second-line anti-TB drugs in Sudan.

### What is known about this topic

- The assay has moderate test sensitivity for the detection of fluoroquinolone and second-line injectable resistance, with high test specificity;
- There was significant heterogeneity in the sensitivity for the detection of kanamycin across studies, resulting in the assay being considered to be insufficient;
- Despite high pooled specificity estimates for all second-line drugs evaluated, the lower pooled sensitivity estimates mean that negative results for resistance cannot be considered to reliably rule-out resistance, as rates of false-negative results were variable among the reported studies and quite high for the detection of resistance to kanamycin.

### What this study adds

- Study evaluated the test performance in the local work situation against the local strains and their different mutations;
- Study ensures the quality of the service that is delivered by the local health system.
